# CRTC1::TRIM11 fusion (CMTCT) tumor with novel presentation in the nasal cavity of three patients

**DOI:** 10.1007/s12105-026-01985-1

**Published:** 2026-11-12

**Authors:** Bushra Sayeed, Mark G. Evans, Adam Bedeir, Anthony Crymes, Mark Swanson, Adrian J. Correa, Elías Sobrino-Najul, Camille Santiago-Negrón, María Correa-Rivas, Jeffson Chung, Meghan Turner, Lakshmi Garladinne, Curtis Johnston, Matthew J. Oberley, Hiba Qari, John Ozolek

**Affiliations:** 1 Department of Pathology, Anatomy, and Laboratory Medicine, West Virginia University, Morgantown, WV USA; 2 Caris Life Sciences, Phoenix, Arizona USA; 3 Basis Phoenix High School, Phoenix, Arizona USA; 4 Keck School of Medicine, University of Southern California, Los Angeles, California USA; 5 Hospital Oncológico Dr. Isaac González Martínez, San Juan, Puerto Rico; 6 University of Puerto Rico, San Juan, Puerto Rico; 7 Department of Otolaryngology/Head and Neck Surgery, West Virginia School of Medicine, Morgantown, WV USA; 8 Department of Radiology, West Virginia University School of Dentristy, Morgantown, WV USA; 9 University of Washington School of Dentistry, Oral Pathology, Seattle, Washington USA; 10 Indiana University and Indiana University Health Department of Pathology, Indianapolis, Indiana USA

**Keywords:** CRTC1:TRIM11 fusion, Sinonasal, Neuroendocrine, Melanocytic, Clear cell sarcoma

## Abstract

**Purpose:**

CRTC1::TRIM11 cutaneous melanocytic tumors are a recently described uncommon entity almost exclusively reported as arising in the skin with fewer than 70 described cases and no dedicated reports of tumors arising in the sinonasal cavity (SNC). We identified 3 cases arising in the SNC with similar morphology and immunophenotype, all harboring CRTC1::TRIM11 fusion. We present the clinical, histological, immunophenotype, and molecular profile of these tumors and compare our tumors with those previously reported in the literature and provide practical guidance for evaluating tumors in the SNC with undifferentiated, neuroendocrine, or melanocytic phenotype.

**Methods:**

Formalin-fixed, paraffin embedded tissue sections of the patients’ tumors were examined and compared with routinely stained sections and by immunohistochemistry. Whole exome/whole transcriptome sequencing revealing CRTC1::TRIM11 fusions in each case was performed at Caris^®^ Life Sciences.

**Results:**

The patients were 34, 48, and 69 years of age, 2 females and 1 male. All tumors originated in the nasal cavity and hard palate. Original diagnoses were S100-negative granular cell tumor, poorly differentiated carcinoma and mucosal melanoma. All tumors demonstrated highly cellular proliferations of round/ovoid cells arranged in vague organoid clusters. Individual cells had moderate eosinophilic granular cytoplasm and round nuclei containing dispersed and granular chromatin with visible nucleoli resembling neuroendocrine tumors. The neural markers synaptophysin and chromogranin were negative, however, tumors expressed PGP9.5 and NCAM in 2 cases. All cases expressed SOX10 in a diffuse fashion but negative for S100, Melan-A, HMB-45. CRTC1::TRIM11 fusion was detected in all cases.

**Conclusions:**

This recently described CRTC1::TRIM11 fusion-driven tumor almost exclusively described in cutaneous sites, hence the current designation as cutaneous melanocytic tumor with CRTC1::TRIM11 fusion (CMTCT), can arise in the nasal cavity and should be considered in the differential diagnoses of aggressive tumors with similar morphologies in the SNC that include mucosal melanoma, sinonasal undifferentiated carcinoma, and neuroectodermal and neuroendocrine carcinomas.

## Introduction

Cutaneous melanocytic tumor with CRTC1::TRIM11 fusion (CMTCT) was first described in a series of 5 cases in 2018 by Cellier et al. [1]. All cases in this series presented as unpigmented primary intradermal nodular tumors with a melanocytic immunophenotype. All tumors expressed S100, MITF, SOX10 and focally expressed MelanA or HMB45. The differential diagnoses included cutaneous clear cell sarcoma, primary dermal melanoma and paraganglioma-like melanocytic tumors. However, molecular profiling by RNA sequencing identified a CRTC1::TRIM11 fusion later confirmed by fluorescent in situ hybridization (FISH) with TRIM11 breakapart probe. This series distinguished these fusion tumors from other unpigmented nodular tumors with melanocytic phenotype. The tumors in this series demonstrated low-grade behavior with no recurrence after wide local excision with negative margins at a median follow-up period of 14 months. Subsequently, a case with metastasis was described by Bontoux et al. that was also previously described as clear cell sarcoma, but it was later diagnosed as CMTCT due to the presence of the CRTC1::TRIM11 fusion [2]. Based on this, it was considered to potentially have more aggressive biological potential and thus termed cutaneous melanocytic tumor with CRTC1::TRIM11 fusion. Following this Ko et al. described four additional cases on the leg, ear and face that were uniformly negative for melanocytic markers but uniformly expressed SOX10 by IHC [3]. To date, 67 cases have been published in the medical literature (all languages; 64 English, 3 Chinese) of CRTC1::TRIM11 fusion tumors with the vast majority presenting as cutaneous or dermal nodules with only 2 cases occurring in unspeified “mucosal” areas [4], and one recent bona fide presentation in the bone [5]. The differential diagnoses in these cases prior to the discovery of a shared CRTC1::TRIM11 fusion have included several entities; conventional nevi, cellular neurothekeoma, granular cell tumor, Spitz nevi, melanocytoma, melanoma, clear cell sarcoma, schwannoma, and epithelioid malignant peripheral nerve sheath tumors. More recently, in a 2025 review of fusion-associated cutaneous tumors with melanocytic differentiation, Ko has described several recognized fusion tumors that are dermal-based with melanocytic differentiation [6]. These tumors are clear cell sarcoma (CCS), clear cell tumor with melanocytic differentiation and ACTIN::MITF (CCTMAM), clear cell tumor with melanocytic differentiation and MITF::CREM (CCTMMC), cutaneous tumor with MED15::ATF1 (CTMA), and the CRTC1::TRIM11 cutaneous tumor (CTCT). While these CRTC1::TRIM11 fusion tumors have been described in the head and neck site, they have all been “typical” with presentation in the dermis. To date, no cases of tumors harboring the CRTC1::TRIM11 fusion have been described occurring in the SNC. We present 3 cases of tumors harboring the CRTC1::TRIM11 fusion presenting in the SNC. We review their clinical findings, histology, immunoprofile, and review the literature with comparison to those tumors already described and include this tumor in the differential diagnosis of sinonasal tumors.

## Methods

### Immunohistochemistry (IHC)

IHC was performed on 4-5-mm thick formalin-fixed paraffin embedded (FFPE) tissue sections per manufacturer instructions and were interpreted by board-certified pathologists (MGE, AJC, MCR, JO) in the context of appropriate positive and negative controls. Markers included here were: SOX10, S100, HMB45, Mart-1/MelanA, smooth muscle actin, cytokeratin AE1/3, CD56, PGP9.5, and synaptophysin.

### Molecular

All genomic testing was performed by Caris^®^ Life Sciences.

### DNA Sequencing

Genomic DNA was isolated from FFPE tumor samples and underwent whole exome sequencing utilizing Illumina NovaSeq technology (San Diego, CA) and a customized Agilent SureSelect Human All Exon V7 bait panel (Santa Clara, CA) with high read depth for over 700 clinically relevant genes, along with enrichment for another 20,000 + genes at lower depth.

### RNA Sequencing

Gene fusion detection was completed by whole transcriptome sequencing performed on FFPE mRNA extracted using the Agilent SureSelectXT Low Input Library prep chemistry (Santa Clara, CA) and analyzed with Illumina NovaSeq. Raw data was demultiplexed by Illumina Dragen BioIT accelerator, trimmed, counted removing PCR-duplicates, and aligned to human reference genome GRCh37/hg19 by STAR aligner. Gene fusions supported by ≥ 3 junction reads were reported.

## Case Reports

### Case 1

**Fig. 1:**
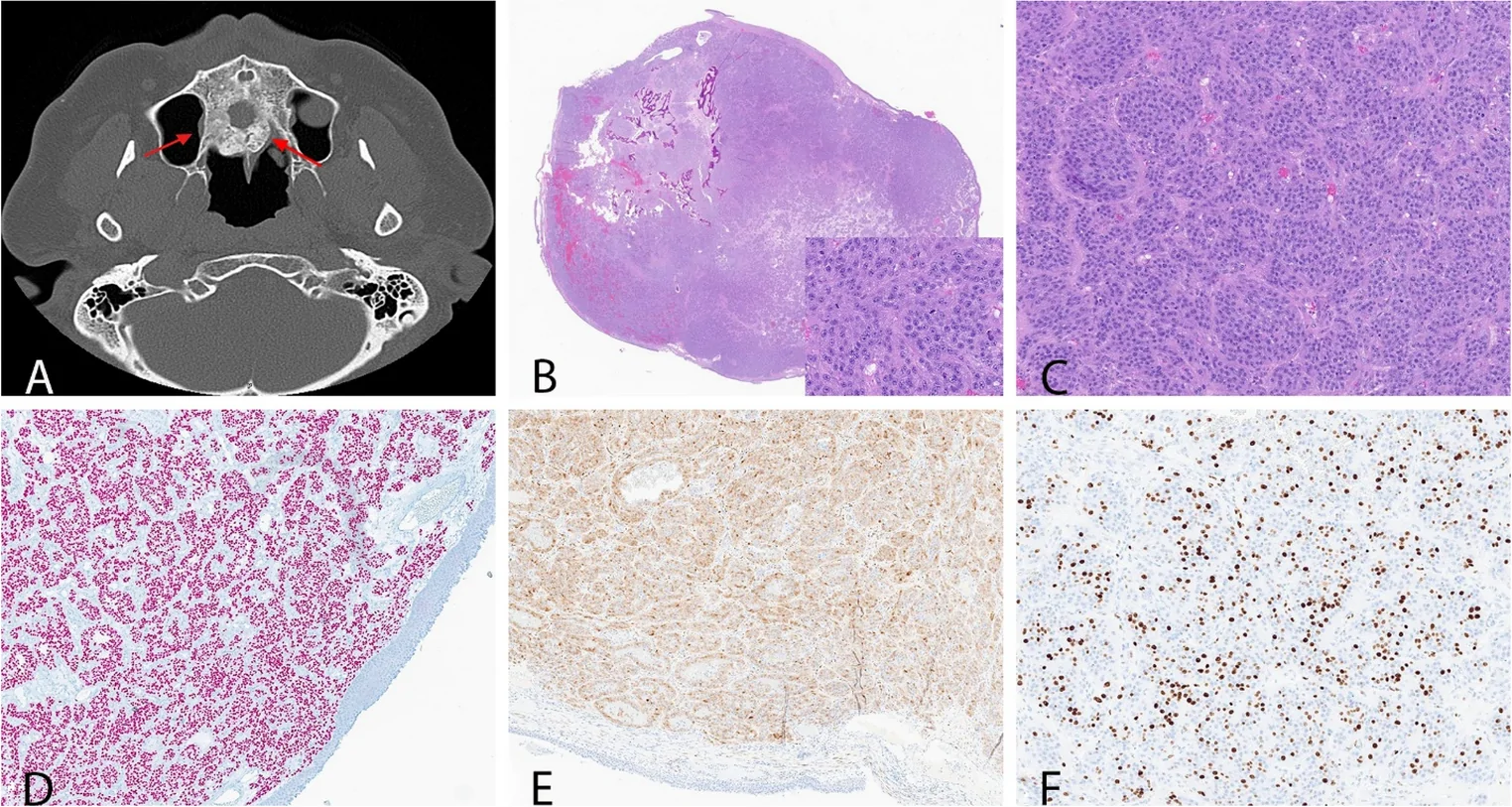
Case 1: (**A**) CT of the nasal mass showing and expansile, destructive lesion filling the nasal cavity with involvement of the hard palate (arrows). **B **Low magnification view of the large nasal mass with extensive bony involvement (HE, 5X). Inset shows vague organoid microarchitecture and relatively uniform round-ovoid cells with “neuroendocrine” type chromatin (HE, 400X). **C **Organoid architecture separated by thin hyalinized tissue punctuated by small vessels (HE, 100X). **D **SOX10 staining nuclei diffusely (SOX10, 50X). **E **Diffuse cytoplasmic and focal nuclear staining with PGP9.5 neuroblastic marker (PGP9.5, 50X). **F **Ki-67 shows a high proliferation index (30% or higher) (Ki-67, 100X)

The patient was a 48-year-old male who presented with a chief complaint of a slowly growing midline hard palate mass, affecting speech, present for more than 8 years. MRI of the facial bones demonstrated a soft tissue mass expanding the bony hard palate with thinning and erosion (Fig. 1A, See Tables 1 and 2 for clinical and IHC data). Biopsy of the hard palate lesion was ultimately reported as S100-negative granular cell tumor after thorough evaluation and outside consultation. Tumor debulking was performed with margins involved. After multiple recurrences, a left medial maxillectomy, revision septectomy, left ethmoidectomy and middle turbinate excision were performed with tumor present at one margin. The specimen from the medial maxillectomy demonstrated a 6.0 cm well-circumscribed tan-gray lobulated mass. Microscopically, the tumor showed a highly cellular proliferation of round/ovoid cells arranged in vague organoid clusters. The cells had high nuclear-to-cytoplasmic ratio with round nuclei containing “salt and pepper” chromatin, occasional nucleoli, and indistinct eosinophilic granular cytoplasm (Fig. 1B and C). Additionally, numerous apoptotic and mitotic figures, approximately 3–5 per HPF (approximately 40 per 10 HPF) were identified. The tumor overall closely resembled a neuroendocrine-type neoplasm and more extensive evaluation was performed to exclude this possibility. Notably, this tumor diffusely expressed SOX10 (Fig. 1D), NCAM (CD56), galactin-3, PGP9.5 (Fig. 1E), p53, vimentin and beta-catenin. The tumor did not express cytokeratins (AE1/AE3, CK 5/6, CK 8/18, CK 20), EMA, p63, synaptophysin, chromogranin, NKX3.1, smooth muscle actin, desmin, CD 68, CD163, calretinin, HMB-45, Mart-1/Melan-A, S-100. A Ki-67 proliferation index was approximately 30% (Fig. 1F). Whole exome/whole trancriptome sequencing detected a CRTC1::TRIM11 fusion by RNA sequencing and TERT mutation (pathogenic variant, c.-124 C > T) by DNA sequencing. BRAF, NTRK 1/2/3, and EWSR1 mutations were not identified.

**Table 1:** Clinical data

Case	Age	Sex	Site	Size (cm)	Initial diagnosis	Original excision	Re-excision	Recurrence	Follow-up (mos)
1	48	M	Hard palate	6	S100-negative granular cell tumor	Incomplete	Yes +	Yes	21
2	34	F	Right nasal vestibule	3	Poorly differentiated carcinoma	Unknown	Yes	Yes	120
3	69	F	Right nasal cavity	3	Mucosal melanoma	Complete	Yes	No	16

+ positive margins

**Fig. 2:**
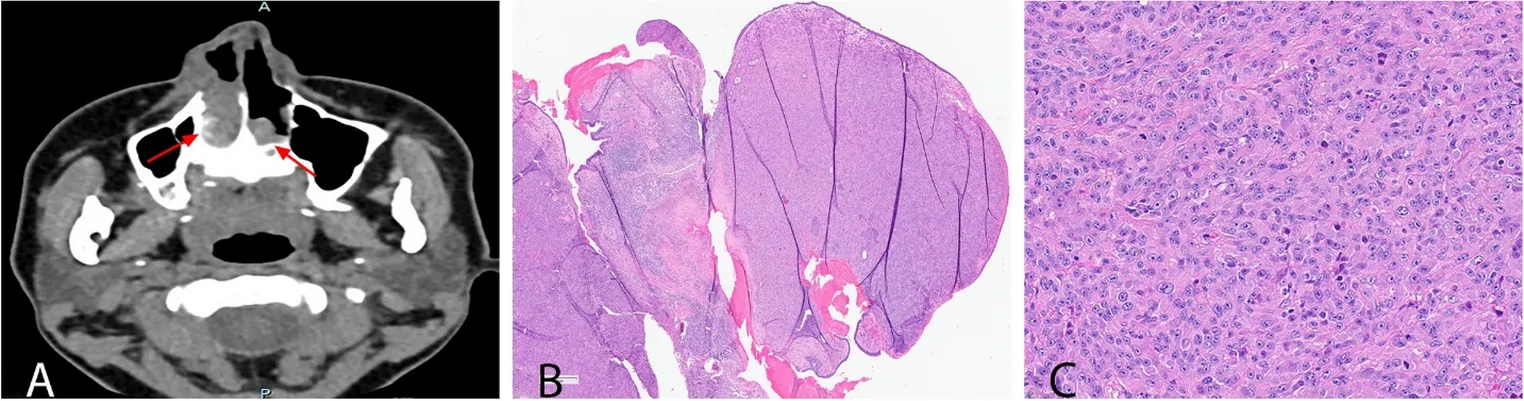
Case 2: (**A**) CT of recurrent tumor showing 3.0 cm recurrence of the nasal cavity tumor (arrows) that was subsequently resected. **B **Low magnification of the nodular and cellular tumor (HE, 5X). **C **Higher magnification showing a tumor composed of a dense nodular infiltrate of round-to-ovoid cells with eosinophilic cytoplasm and nuclei with open chromatin and single prominent nucleoli and less than 5 mitoses per 10 high-power fields (HE, 200x)

### Case 2

This patient was a 34-year-old woman with a medical history of a poorly differentiated carcinoma of the right nasal vestibule status post right partial rhinectomy with ear cartilage reconstruction. Subsequently, she underwent radiation therapy completing 10 of 30 prescribed treatments and an unspecified number of chemotherapy cycles. One year later, she presented with a local recurrence (tumor involving the right inferior turbinate, right nasal floor, nasal septum, and left nasal floor) and right neck metastasis (Fig. 2A, See Tables 1 and 2 for clinical and IHC data). The patient subsequently underwent a medial maxillectomy, septoplasty, inferior parotidectomy, and right modified radical neck dissection (levels 1B, 2A, 2B, 3 and 4.) Tumor was identified in 4 of 43 dissected neck lymph nodes. The tumor was composed of a dense nodular infiltrate of round-to-ovoid cells with eosinophilic cytoplasm and nuclei with open chromatin and single prominent nucleoli with less than 5 mitoses were appreciated per 10 high -power fields (Fig. 2B and C). By immunohistochemistry, the neoplastic cells diffusely expressed SOX10 and MITF and did not express pancytokeratin, synaptophysin, SSTR2, estrogen receptor, progesterone receptor, SMA, STAT6, clusterin, CD10, CD21, CD30, CD31, CD 34, CD 35, CD 163, ALK, MUM-1, GFAP, HMB-45, Mart-1/Melan-A, and S100. INI1 expression was retained. Molecular testing confirmed the presence of CRTC1::TRIM11 fusion with an accompanying TERT mutation (c.-146 C > T).

**Table 2:** Immunohistochemical staining results:

Case	SOX10	S100	HMB45	Mart-1/MelanA	SMA	AE1/3	CD56	PGP9.5	Synaptophysin
1	Positive	Negative	Negative	Negative	Negative	Negative	Positive	Positive	Negative
2	Positive	Negative	Negative	Negative	Negative	Negative	Negative	Not done	Negative
3	Positive	Patchy	Patchy	Patchy	Not done	Negative	Not done	Not done	Negative

### Case 3

**Fig. 3:**
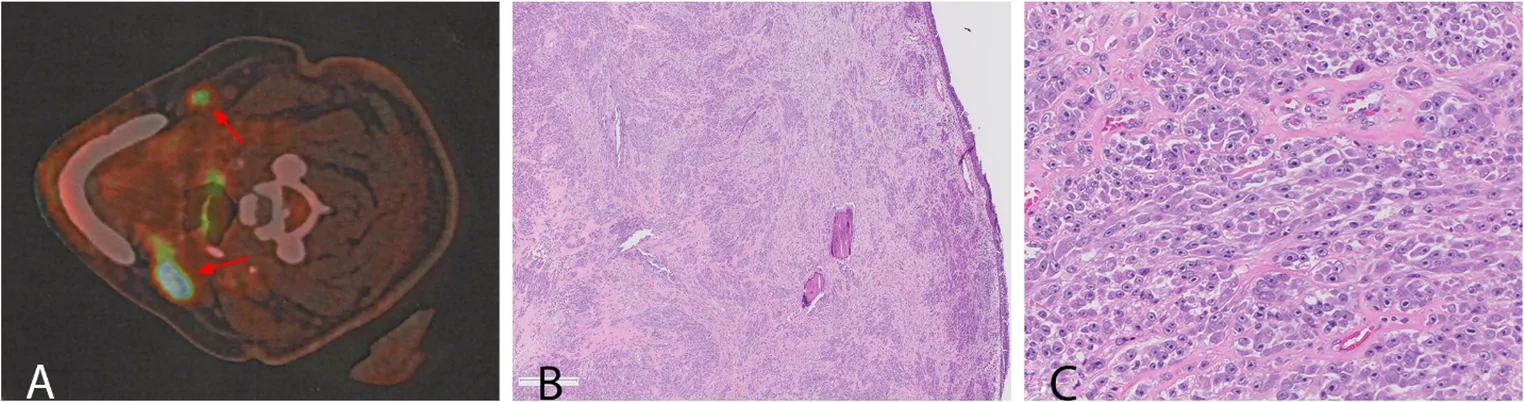
Case 3: (**A**) Post-operative PET/CT demonstrated small hypermetabolic nodules of the right anterior neck and submandibular regions (arrows). **B **Lower magnification view of original tumor showing nests of cells within a somewhat hyalinized stroma (HE, 5X). **C** Higher magnification shows nests and cords of round-to-ovoid cells with eosinophilic cytoplasm. The nuclei were predominantly vesicular with a single prominent nucleolus. Mitotic activity was low (less than 5 mitoses per 10 HPFs) (HE, 200X)

The patient was a 69-year-old Latina woman with a past medical history that included atherosclerotic cardiac disease, diabetes mellitus and hypertension. She presented with a four-month history of recurrent right sided epistaxis and was found to have a 3.0 cm right nasal cavity mass on physical examination. The tumor was excised with positive margins, and a diagnosis of nasal mucosal melanoma was rendered. Post-operative PET/CT demonstrated small hypermetabolic nodules of the right anterior neck and submandibular regions (Fig. 3A). The patient’s original nasal tumor consisted of nests and cords of round-to-ovoid cells with eosinophilic cytoplasm. The nuclei were predominantly vesicular with a single prominent nucleolus. Mitotic activity was low, with less than 5 mitoses per 10 high-power fields (Fig. 3B, C). By immunohistochemistry, SOX10 was strongly expressed in the majority of tumor cells with scattered tumor cells expressing Mart-1/Melan-A, HMB-45 and S100. Neuroendocrine markers synaptophysin and CD56 as well as pancytokeratin were negative. Molecular testing confirmed the presence of CRTC1::TRIM11 fusion without other mutations present.

## Discussion

To date, 67 cases of tumors having melanocytic differentiation harboring the CRTC1::TRIM11 fusion, termed cutaneous melanocytic tumor with CRTC1::TRIM11 fusion (CMTCT) or more recently, CRTC1::TRIM11 cutaneous tumor (CTCT), have been reported since first described in the literature in 2018 [1–5, 7–10]. Of these cases, two have been noted to occur in unspecified “mucosal” sites and none within the SNC. Here we describe the first three cases in the literature of tumors showing melanocytic differentiation with CRTC1::TRIM11 fusion occurring specifically within the SNC.

The CRTC1::TRIM11 fusion involves a chromosomal rearrangement in exon 1 of CREB-regulated Transcription Coactivator 1 (CRTC1) and exons 2–6 in Tripartite Motif-Containing 11 (TRIM11) [t(19;1)(p13.11;q42.13)] [10]. It is fusion of 5’ portion of the CRTC1 gene on 19p13 and the 3’ end of the TRIM11 gene on 1q42. Two cases have shown the breakpoint occurring in exon 2 of CRTC1 resulting in a similar in-frame fusion protein [4]. TRIM11 is an E3 ubiquitin ligase. It is known to enhance the degradation of misfolded proteins by tumoral cells to promote tumorigenesis. It has been described in cancers such as gliomas, colon, lung and liver cancers and has been associated with worse prognosis [1]. However, the fusion protein is predicted to lack this activity and may need further evaluation to determine its function [4]. CRTC1 is involved in the cell-cycle as a cAMP response element-binding protein (CREB). Translocation of CRTC1 gene is also observed in other tumors like clear cell hidradenoma, mucoepidermoid carcinoma and Warthin tumors [1]. Immunohistochemistry for TRIM11, although not readily performed in most laboratories, can be performed with most cases expressing this marker in similar fashion to SOX10 [4].

From a clinical perspective, briefly, this fusion tumor was originally thought to be an indolent tumor when first reported, 8 of the 54 cases to date have noted recurrence and 7/8 with lymph node metastasis including 5 with pulmonary metastatic disease [1–4, 7–12]. And while most tumors show no aggressive histological features, tumors with high mitotic rates (> 5/hpf), lymphovascular invasion, and additional molecular alterations may portend higher risk for more aggressive clinical behavior [6, 12]. One case previously described (4) with lymph node and pulmonary metastasis had TERT mutations in addition to the CRTC1::TRIM11 fusion. This combination may have predisposed to the more aggressive clinical behavior [12]. More recently, Trichy et al., in their meta-analysis of all reported cases (*n* = 62) of CRTC1::TRIM11 fusion tumors demonstrated 12 cases with metastases with 3/9 cases harboring a TERT mutation which was statistically significant when compared to genomic findings in non-metastatic cases. Average mitotic count and tumor diameter were also significantly higher in metastatic cases [13]. In our small series, two had recurrence (Cases 1 and 2) and follow-up was not available in one case. TERT mutation was also detected in Case 1 and Case 2, with Case 1 demonstrating high proliferation index (> 30%) and mitotic count. The patient described in Case 1 had multiple recurrences. Case 1 demonstrated a very high Ki-67 proliferation index exceeding 30%. as well as Cases 1 and 2.

The SNC is beginning of the upper aerodigestive tract lined by squamous and respiratory type ciliated mucosa depending on the specific anatomic location. It begins at the nasal vestibule with medial, lateral and superior boundaries of the nasal septum, turbinates, and skull base respectively and encompassing the paranasal sinuses (maxillary, frontal, ethmoid, sphenoid). A host of tumors within the differential diagnoses of “melanocytic” tumors can arise in the SNC including undifferentiated carcinomas (e.g. sinonasal undifferentiated carcinoma), tumors with neuroendocrine differentiation (e.g. olfactory neuroblastoma, small and large cell neuroendocrine carcinomas), and tumors with melanocytic differentiation (e.g. mucosal melanoma). The CRTC1::TRIM11 fusion tumor can now be added to the list of entities that should be considered when confronted by tumors with undifferentiated, neuroendocrine, or melanocytic phenotypes arising in the SNC.

The histologic features of CMTCT or CTCT have been uniformly described as nests and fascicles of spindled to epithelioid cells with pale pink to vacuolated cytoplasm, prominent nucleoli, variable cytologic atypia, vaguely neural-like appearance, intervening fibrous/hyalinized bands and mitotic activity typically < 5/hpf. Perineural invasion, lymphovascular invasion and ulceration are typically absent [4] The age cohort is fairly wide, spanning 5 to 82 years of age, but predominantly in middle-aged adults [1, 3, 10]. The one consistent marker by IHC in all tumors is the expression of SOX10, a marker for neural crest and melanocytic differentiation, and TRIM11. Not only is SOX10 expressed in all tumors but invariably expressed in a diffuse fashion. All tumors in our series also diffusely expressed SOX10. S100 has been reported to be expressed in perhaps 40–50% of cases and HMB-45 or MelanA is only focally expressed or negative as well as little to no expression of keratins or neural markers such as synaptophysin [1–4, 7–12]. The cases in our series demonstrated negative or negligible expression of S100 and were negative for melanoma markers Melan-A and HMB-45, keratin, and synaptophysin as might be expected based on other reported cases. The significant expression of PGP9.5, a neuroblastic marker, in Case 1 is interesting and has not been reported in other cases.

Within the pathological differential diagnoses of SNC tumors, epithelial lesions including sinonasal undifferentiated carcinoma, lymphoepithelial-like carcinoma, NUT midline carcinoma, sinonasal neuroendocrine carcinoma, can demonstrate sheets of monotonous small-to medium sized cells as seen in the CRTC1::TRIM11 fusion tumors. However, they uniformly stain positive for pan-cytokeratins, unlike CMTCT [14]. Epithelioid schwannoma and epithelioid malignant peripheral nerve sheath tumors have epithelioid morphology similar to CMTCT but typically express EMA, low-molecular weight cytokeratins, calponin and p63.

Melanomas rarely exhibit round cell morphology typically expressing MART-1/MelanA, HMB45, SOX10 and S100 in a generous fashion. Mucosal melanomas histologically are very similar to CMTCT with epithelioid and spindled cells arranged in a nested fashion with melanin noted in some cases. However, the IHC profile typically shows expression of S100, HMB45, and MelanA/MART1 [14]. The molecular profile in sinonasal melanomas differs from cutaneous melanomas showing NRAS mutations over BRAF, which are characteristic of cutaneous melanomas [11, 15]. To date, BRAF mutations, specifically, BRAFV600E have not been reported in CRTC1::TRIM11 fusion tumors. The morphology and expression of SOX10 makes distinguishing the CRTC1::TRIM11 fusion tumor from mucosal melanoma difficult and invoking molecular testing as appropriate. The focal or lack of expression of Melan-A and HMB-45 in the CRTC1::TRIM11 fusion tumors would certainly favor melanoma.

Sarcomas, such as, clear cell sarcoma (CCS), rhabdomyosarcoma, desmoplastic round cell tumor, poorly differentiated synovial sarcoma, mesenchymal chondrosarcoma and small cell osteosarcoma and Ewing sarcoma may clinically present similarly in the SNC. Of these entities, clear cell sarcoma has been the one most associated in the differential diagnosis of CMTCT in the cutaneous setting [6, 8]. CCS would appear as nests of spindled and epithelioid cells surrounded by collagen fibers, with interspersed wreath-like giant cells, which are different from the multinucleated giant cells that may be present in CMTCT. The IHC profile can mimic CRTC1::TRIM11 fusion tumors with expression of SOX10, S100, HMB45 and MelanA. Again, molecular characterization would be appropriate here as the CCS typically harbors the EWSR1-ATF1 fusion gene, distinguishing it from CRTC1::TRIM11 fusion tumors [11]. Other rare tumors in the SNC including rhabdomyosarcoma occur most frequently in the pediatric population and notably expresses desmin, myoD1 and myogenin. Poorly differentiated synovial sarcoma may express pan-cytokeratin and shows SS18 gene rearrangement. Mesenchymal chondrosarcoma and small cell osteosarcoma require the identification of matrix for diagnosis [14]. Ewing sarcoma, another malignant tumor that harbors EWSR1 gene rearrangements, can occur in the SNC rarely and shows undifferentiated primitive small round blue cells without a significant stromal component and notable for diffuse expression of CD99 in a membranous fashion [14].

The initial “gestalt” impression upon evaluation of the tumor resection in Case 1 was that of a neuroendocrine tumor. This was supported by two observations: (1) vague organoid/Zellballen microarchitecture and (2) nuclear features including the chromatin pattern (“salt and pepper”). These observations did not seem to adequately fit with the initial diagnosis of “S100-negative granular cell tumor” based on the limited descriptions of this very rare entity [16, 17]. Other neuroendocrine tumors presenting in the SNC include neuroendocrine carcinoma (NEC), that encompases atypical carcinoid, carcinoid, small cell poorly differentiated, large cell poorly differentiated tumors. Poorly differentiated NEC exhibits diverse architectural patterns, varying in cell shape, size and cytoplasmic features. IHC expression is notably positive for pan-cytokeratin and neuroendocrine markers like CD56/NCAM, synaptophysin, chromogranin which are typically not expressed or expressed in very limited fashion in CRTC1::TRIM11 fusion tumors. Neuroectodermal tumors are a group mostly represented by olfactory neuroblastoma (ONB) (14). ONB represents 6% of the sinonasal tumors and like NEC consistently expresses neural markers; synaptophysin, chromogranin A, neurofilaments, and CD56 [15, 18, 19].

## Conclusion

Our series highlights three cases of CRTC1::TRIM11 fusion tumor occurring in the sinonasal cavity; a site not previously encountered in the clinical spectrum of these tumors. This tumor needs to be considered in head and neck tumors showing undifferentiated, neuroendocrine or melanocytic phenotype. The IHC panel to broadly classify the tumors with histology as noted above should include but not limited to cytokeratins, SMA, MelanA/MART-1, HMB-45, SOX10, S100, CD56 and synaptophysin. A tumor diffusely expressing SOX10 with very little to no expression of the other markers should prompt molecular testing for CRTC1::TRIM11 fusion.

## Data Availability

No datasets were generated or analysed during the current study.

## Declarations

3) This research does not contain any studies with human participants performed by any of the authors.

4) This study does not contain any studies with animals performed by any of the authors.

5) The present study was conducted in accordance with guidelines of the Declaration of Helsinki, Belmont Report, and U.S. Common Rule. With compliance to policy 45 CFR 46.101(b), this study was conducted using retrospective, de-identified clinical data, and patient consent was not required. For this study, consent for publication was not required.
